# Assessing the effectiveness of low-enthalpy geothermal energy for greenhouse temperature regulation towards enhancing desert agriculture

**DOI:** 10.1038/s41598-025-22294-7

**Published:** 2025-11-07

**Authors:** Anwar Hegazy, Ajit Govind

**Affiliations:** 1 Department of Mechanical Engineering, Arab Academy for Science, Technology and Maritime Transport, Alexandria, 1029 Egypt; 2 International Center for Agricultural Research in the Dry Areas (ICARDA), Cairo, 11728 Egypt

**Keywords:** Greenhouse, ERA5-Land, Sustainable agriculture, Arid land, Geothermal energy, EAHE, GIS, Climate sciences, Energy science and technology, Engineering, Environmental sciences

## Abstract

This study investigates the viability and potential of the Earth-Air Heat Exchanger (EAHE) low-enthalpy geothermal system for greenhouse climate control in arid regions, specifically addressing the prevalent challenge of limited meteorological data. Our approach integrates ERA5-Land data with a subsurface soil temperature model, enabling accurate EAHE design and performance prediction in data-scarce environments like Bahariya Oasis, Egypt. The research confirmed the significant thermal stability of the subsurface soil, establishing its potential as a consistent heat source/sink. Initial simulations highlighted effective winter heating but revealed a need for enhanced summer cooling. We demonstrated that optimizing the EAHE system by increasing airflow successfully maintained greenhouse temperatures within near-optimal ranges (below 35$\,{}^{\circ }$C in summer, above 20$\,{}^{\circ }$C in winter) throughout the year. This achievement validates EAHE’s effectiveness for dual heating and cooling in extreme climates. This work provides a robust, data-driven methodology for designing and implementing sustainable, climate-controlled greenhouses in challenging arid zones.

## Introduction

Greenhouse cultivation offers significant advantages over traditional open-field farming by providing a controlled environment that optimizes crop growth. This controlled setting protects crops from adverse weather conditions, such as frost, excessive heat, strong winds, and heavy rainfall, leading to more consistent and reliable yields^1^. This results in higher quality produce, increased yield per square meter, and the flexibility to cultivate a wider variety of crops, off-season produce, expanding market opportunities and ensuring year-round availability^1^. Furthermore, controlled irrigation systems, commonly employed in greenhouses, drastically reduce water wastage compared to traditional field irrigation, making greenhouse farming a more sustainable and water-efficient agricultural practice^2^. In arid regions, a primary challenge for greenhouse cultivation is maintaining optimal indoor climate conditions, specifically ventilation rate, temperature, and relative humidity^3^. Given the elevated ambient temperatures, research on greenhouses in these areas has focused on developing sustainable cooling strategies^4^. Ghani et al.^3^ have highlighted various approaches, while a recent review by Soussi et al.^5^ thoroughly discusses greenhouse interior condition control technologies.

Maintaining optimal temperatures within a greenhouse requires significant energy input for both heating and cooling^1^. Cooling typically relies on natural ventilation through vents, forced ventilation using exhaust fans, or evaporative cooling systems that reduce air temperature through water evaporation^4^. The energy required for these systems depends on factors such as greenhouse size, desired climate conditions, external weather, and the specific crops being cultivated, as different plants have varying optimal temperature and humidity ranges^5^. In arid regions, evaporative cooling is heavily reliant on water, with consumption potentially reaching up to four times the amount of irrigation water^6^. Therefore, in water-scarce regions, research has increasingly focused on minimizing water consumption, identifying sustainable alternatives like geothermal energy^7^. A study by Hegazy et al.^4^ demonstrated geothermal energy as a reliable, non-water-intensive sustainable alternative. The integration of low-enthalpy geothermal energy in greenhouses located in arid regions has been the subject of numerous investigations. Studies conducted in countries like Egypt^8^, Australia^9^, Algeria^10^, and Saudi Arabia^11^ have consistently highlighted the significant potential of low-enthalpy geothermal systems to deliver substantial energy and water savings in agricultural settings.

A crucial step in evaluating the effectiveness of a low-enthalpy geothermal system involves accurately determining the sub-soil temperature at the chosen installation site^7^. This can be achieved through on-site field measurements^12^ or mathematical modeling techniques^13^. While experimental approaches offer direct data, their practical applicability may be limited due to cost and logistical considerations, underscoring the need for more analytical and cost-efficient methods^7^. Mathematical modeling, which typically leverages transient heat conduction, differential equations and energy balance equations at the ground surface, has been widely applied to address this need^14^. Despite the utility of mathematical modeling, simulating the ground’s thermal behavior as a function of both depth and time presents considerable challenges^15^. These complexities arise from fluctuating weather conditions, seasonal variations, varying soil moisture content, and inconsistent thermal conductivity^15^. To overcome these difficulties, Ozgener et al.^15^ proposed a practical and reliable approach to predict daily soil temperature variations by estimating temperatures based on depth and time, utilizing readily accessible data such as annual daily average air temperatures. This approach has gained widespread acceptance and is commonly used in the field^16^. Hegazy et al.^4,8,9,17^ utilized Typical Meteorological Year (TMY) data to estimate sub-soil temperatures in arid regions. However, this method relies on the availability of nearby weather stations to provide the necessary climatic information^7^. A significant hurdle in harnessing low-enthalpy geothermal energy, especially in arid regions, is the limited availability of crucial data^7^. As noted by Abdel-Ghany et al.^12^ and Ceglia et al.^18^, areas with restricted access to climate and environmental information face particular challenges in utilizing this energy source.

Recently, Hegazy and Mohamed^7^ used remote-sensing data to unlock sub-surface temperature profile data, highlighting the promising role of satellite data in gathering information for remote lands. Their study assessed the cooling and heating potential of a single piped heat exchanger. When integrating such a system with a greenhouse, multiple pipes connected in parallel should be implemented, similar to the approach by Hegazy et al.^4,8^, who used TMY data to calculate heating and cooling loads. Their study indicated that a higher number of connected pipes brings the interior temperature closer to the ideal range. However, those studies did not consider land availability, excavation cost, the cost of pipes and installation, besides their reliance on the availability of weather data at the site location, which is often not the case in vast arid regions. While low-enthalpy geothermal energy boasts low running and maintenance costs, it involves a high initial capital investment^19^. The primary costs are associated with the number of heat exchanger pipes, pipe installation layout, excavation, and fan size^20^. All these factors require careful calculation to ensure the feasibility of a low-enthalpy geothermal energy solution for greenhouse applications. To achieve this, predictions of the coupled operation of the greenhouse structure and the low-enthalpy geothermal system must be performed under the specific weather conditions of the installation site.

### Scope and objectives

As highlighted in the existing body of literature, comprehensive local meteorological data, essential for accurate greenhouse system design, is frequently unavailable. While low-enthalpy geothermal energy systems have been recognized for their potential in greenhouse climate regulation, their practical implementation and optimization in data-limited arid settings remain largely unexplored, due to limited information on subsurface temperature profile data. While models for sub-soil temperature prediction is a solution, they depend on readily accessible weather station data, which is not consistently available across extensive remote arid zones.

This study aims to address these critical knowledge gaps by developing an integrated methodology. This approach leverages ERA5-Land weather data for sub-surface temperature profiling, thereby enabling a more accurate and robust design of low-enthalpy geothermal systems for greenhouses in data-scarce arid regions. Our primary objectives for this study are threefold. First, we aim to collect and integrate ERA5-Land surface data with a sub-soil temperature profile model. Second, we will model the thermal performance of a low-enthalpy geothermal known as Earth-Air Heat Exchanger (EAHE) system. Finally, we will simulate the coupled operation of a greenhouse structure and EAHE system under site-specific weather conditions, assessing various configurations to determine the heating and cooling potential of the low-enthalpy geothermal energy.

## Methods

### Study area

The Bahariya Oasis, Egypt, served as the study location for this investigation, offering a representative environment of the expansive Western Sahara. This depression in the Western Desert of Egypt is geographically situated approximately 370 km southwest of Cairo within the Giza Governorate, at coordinates around $28^{\circ }\,21^{\prime}\, 5.36^{\prime\prime}\,\text {N}$ and $28^{\circ }\,51^{\prime}\,44.6^{\prime\prime}\,\text {E}$. The region is notably characterized by its constrained water resources, a critical factor influencing its unique ecological and geological features. The location falls under the hot desert climate (BWh) classification according to the Köppen climate classification system.

### Greenhouse model

The greenhouse model in Ref.^8^ was formulated through the application of an energy balance across its five constituent elements: the cover, indoor air, indoor soil surface, and two sub-soil layers. The energy balance equations for each component are subsequently derived:

- Cover
  1 $$\begin{aligned} \rho _{c}c_{p,c}bA_{c}\frac{dT_{c}}{dt}=\dot{Q}_{solar,c}+\dot{Q}_{rad.,c}^{net}+\dot{Q}_{conv.,i-c}+\dot{Q}_{conv,amb.-c} \end{aligned}$$
- Indoor Air
  2 $$\begin{aligned} \rho _{a}c_{p,a}V_{GH}\frac{dT_{i}}{dt}=\dot{Q}_{inf.}+\dot{Q}_{conv.,c-i}+\dot{Q}_{conv,s-i}+\dot{Q}_{E} \end{aligned}$$
- Indoor soil surface
  3 $$\begin{aligned} \rho _{s}c_{p,s}\Delta z_{0}A_{s}\frac{dT_{s}}{dt}=\dot{Q}_{solar,s}+\dot{Q}_{rad.,c-s}+\dot{Q}_{conv.,i-s}+\dot{Q}_{cond.,s1-s} \end{aligned}$$
- Soil layer 1
  4 $$\begin{aligned} \rho _{s}c_{p,s}\Delta z_{1}A_{s}\frac{dT_{s1}}{dt}=\dot{Q}_{cond.,s-s1}+\dot{Q}_{cond.,s2-s1} \end{aligned}$$
- Soil layer 2
  5 $$\begin{aligned} \rho _{s}c_{p,s}\Delta z_{2}A_{s}\frac{dT_{s2}}{dt}=\dot{Q}_{cond.,s1-s2}+\dot{Q}_{cond.,s3-s2} \end{aligned}$$

**Fig. 1:**
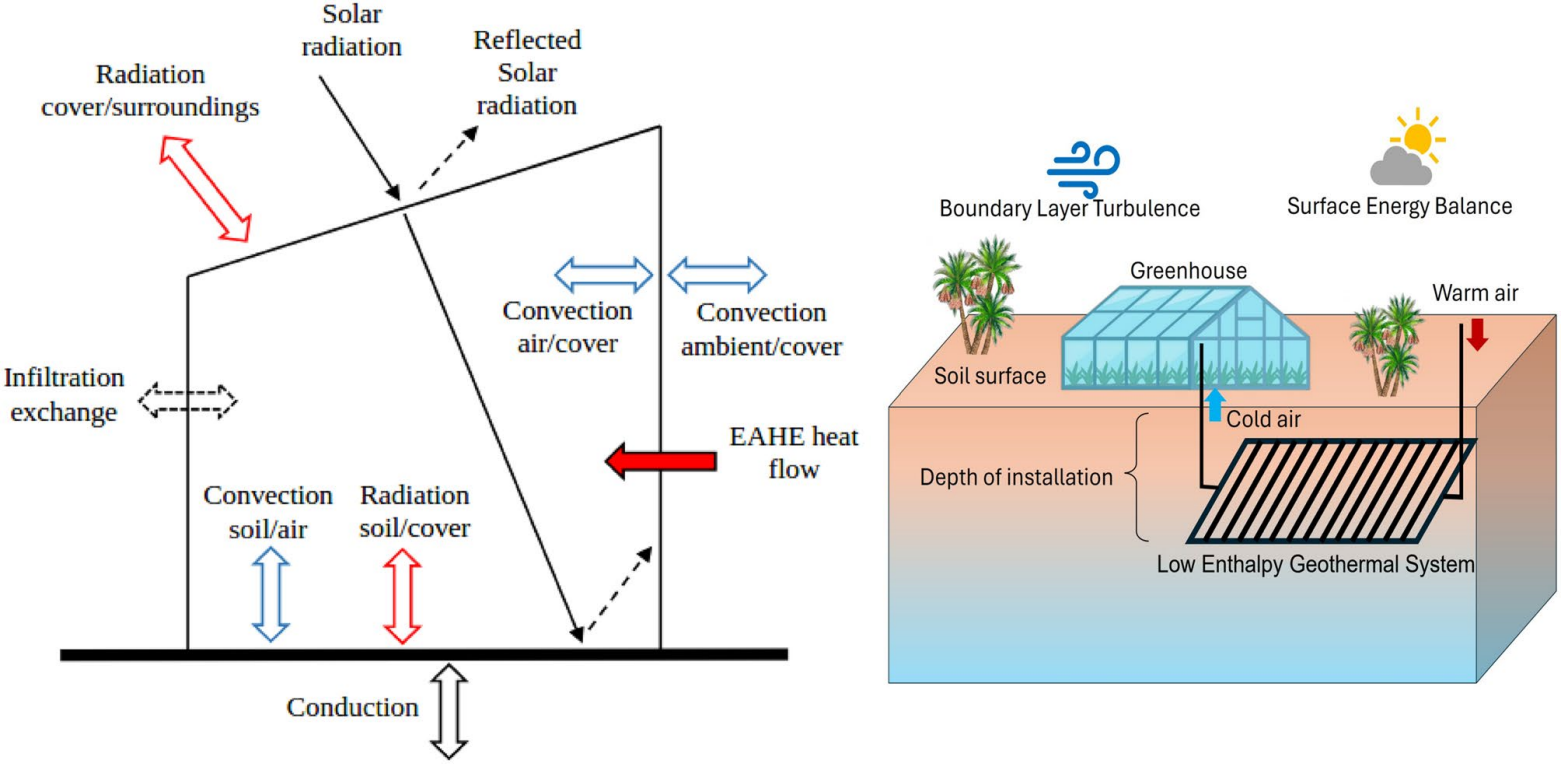
(left) Schematic of the heat flow in the greenhouse model, sourced from Ref.^8^; and (right) schematic of the holistic system. (These schematics are not drawn to scale.).

The heat flows in the above equations are shown in Fig. 1 and are detailed in Ref.^8^. The greenhouse structure size used in our study was 21 $m^3$ and for all the parameters of the greenhouse structure that are not mentioned here, the reader is invited to find them in Ref.^8^. The energy balance equations are ODEs that were solved using MATLAB’s ode45 function. The model was validated against experimental data achieving an Average Absolute Deviation (AAD) of $\pm 1.13$ $\,{}^{\circ }$C in prediction accuracy as shown in Ref.^8^. Therefore, the model prediction accuracy is sufficient for the simulations in our investigation. The schematic of the holistic system is shown in Fig. 1.

### Low-enthalpy geothermal energy EAHE model

The low-enthalpy geothermal energy system employed in this research is an EAHE. This system comprises a configuration of single or multiple pipes laid in parallel beneath the soil surface. Air is actively drawn or forced through these pipes, facilitating a continuous heat exchange process between the circulating air and the surrounding ground. This thermal exchange occurs via two primary mechanisms: convection between the air flow and the inner surface of the pipe wall, and conduction through the pipe material and into the surrounding soil. The convective heat transfer coefficient is a critical parameter in modeling the EAHE’s performance. For laminar flow conditions within the pipes, this coefficient is determined using the following equation $Nu=3.66$ for laminar flow and for turbulent flow the widely accepted Dittus-Boelter correlation is applied $Nu=0.023Re^{0.8}Pr^{n}$ , where the exponent ’n’ is assigned a value of 0.3 for cooling process and 0.4 for heating process^21^. The EAHE system is modeled using the Number of Transfer Units (NTU)-effectiveness method^21^. Within this framework, the EAHE is treated as a constant wall temperature heat exchanger, assuming a consistent sub-soil temperature. Consequently, the EAHE outlet air temperature $T_{E,outlet}$ is calculated using the following equation:

6 $$\begin{aligned} T_{E,outlet}=T_{E,inlet}+(T(z,t)-T_{E,inlet})\times (1-e^{-NTU}) \end{aligned}$$

where, NTU is equal to $\frac{UA_{p}}{\dot{m}_{E}c_{p,a}}$. The value of $T_{E,inlet}$ is the ambient air temperature and the *T*(*z*, *t*) is the soil temperature at depth (z) at the corresponding day (t). Accordingly, since the value $T_{E,outlet}$ then the heat flow $\dot{Q}_{E}$ in Eq. (2) could be calculated. Following recommendation in the studies^4,7,8,17^ the pipe diameter is 0.016 m, pipe length is 50 m and the air speed indise the pipe is 2 m/s and the pipe material considered for the pipe/s was PVC. The EAHE model was validated against experimental data, yielding a prediction accuracy of 2.4%^8^.

### Soil temperature profile with depth

Knowledge of the subsurface soil temperature at the installation depth is a critical input parameter for effectively modeling an EAHE system. However, these data are often not readily available at prospective installation sites. To address this, a model based on heat conduction through a semi-infinite solid can be employed^21^. This model assumes constant soil thermal properties and is used to predict the temperature profile at a specific depth. The model considers that the soil surface temperature experiences a cyclic, annually periodic fluctuation around an annual mean value. This fluctuation can be mathematically represented as $T(0,t)=T_{mean}-\Delta T_{surface}\times \cos \omega (t-t_{o})$ which is applied as a surface boundary condition, while the internal boundary is $T(\infty ,t)=T_{mean}$. Based on these assumptions, the temperature at a given depth (z) for soil with a thermal diffusivity ($\alpha$, in $m^2/day$) can be generated using the following equation^22^:

7 $$\begin{aligned} T(z,t)=T_{mean}-Amp._{surf}\times e^{-z\sqrt{\frac{\pi }{365\alpha }}}\times \cos \left[ \frac{2\pi }{365}\left( t-t_{o}-\frac{z}{2}\left( \sqrt{\frac{365}{\pi \alpha }}\right) \right) \right] \end{aligned}$$

To solve this equation, the values for $T_{mean}$, $\Delta T_{surface}$ and $t_o$ are required. Following the approach outlined in Ref.^7^, these parameters can be approximated from annual weather data. Specifically, $T_{mean}$ , is taken as the mean annual land surface temperature, $\Delta T_{surface}$ is defined as half the difference between the monthly mean soil surface temperatures of January and July, and $t_o$ can be approximated as 36 days. The local soil is characterized as sandy soil^7^, for which the thermal diffusivity ($\alpha$) can be determined from its physical properties. Specifically, the density is 1775 $\text{kg/m}^3$, the specific heat capacity is 840 J/kg K and the thermal conductivity is 0.91 W/mK^21^.

### ERA5-LAND

The meteorological data utilized in this study is sourced from ERA5-Land, a global land-surface reanalysis dataset^23^. This comprehensive dataset is produced by the European Centre for Medium-Range Weather Forecasts (ECMWF) under the auspices of the Copernicus Climate Change Service (C3S). ERA5-Land offers a consistent and high-resolution representation of land surface variable evolution spanning several decades^24^. Hourly meteorological data, including ambient air temperature (AAT), land surface temperature (LST), wind speed, and solar radiation, for the year 2024 were acquired to serve as inputs for the mathematical models. These data drive the greenhouse model, the subsurface temperature profile model, and the EAHE model. Following the calculation of the subsurface soil temperature and the determination of the EAHE installation depth, hourly greenhouse simulations were performed. These simulations were conducted under two scenarios: with and without active temperature control. For the initial hour of each simulation, the ambient air temperature was uniformly set as the initial condition for all modeled components. Subsequently, for all successive hours, the output values from the preceding hour’s simulation were used as the initial conditions for their respective components, ensuring a continuous and dynamic simulation over the entire period.

### Integrated model solution and analysis procedure

We conducted our investigation by using ERA-Land data as inputs for the greenhouse model simulation. We also analyzed ERA-Land data to generate a soil subsurface temperature profile. This temperature profile was then used as an input (boundary condition) for the EAHE model. Following these steps, we performed simulations for the following cases:

- Greenhouse without temperature regulation
- Greenhouse with temperature regulation using EAHE

The predicted greenhouse temperatures from the simulations were analyzed on three levels: hourly throughout the year, as a daily average over the entire year, and over a diurnal cycle for the coldest and warmest days of the year. This comprehensive analysis helps to effectively assess the performance of the low-enthalpy geothermal system for greenhouse temperature regulation.

### Assumptions and limitations

Our analysis is based on the following assumptions:

- To simplify the model, no crops were included in the greenhouse, a choice consistent with similar studies^4,8,25–27^. Practically, it represents the case of the greenhouse seeded or transplanted stages in commercial greenhouses^27^.
- Soil properties are uniform up to 5 m
- To avoid near-pipe soil thermal saturation, which can result from continuous operation and negatively impact EAHE performance, the EAHE pipe is designed with a sufficient length of 50 m.^4^)

## Results and discussion

### Weather data AAT, wind speed and solar radiation

**Fig. 2:**
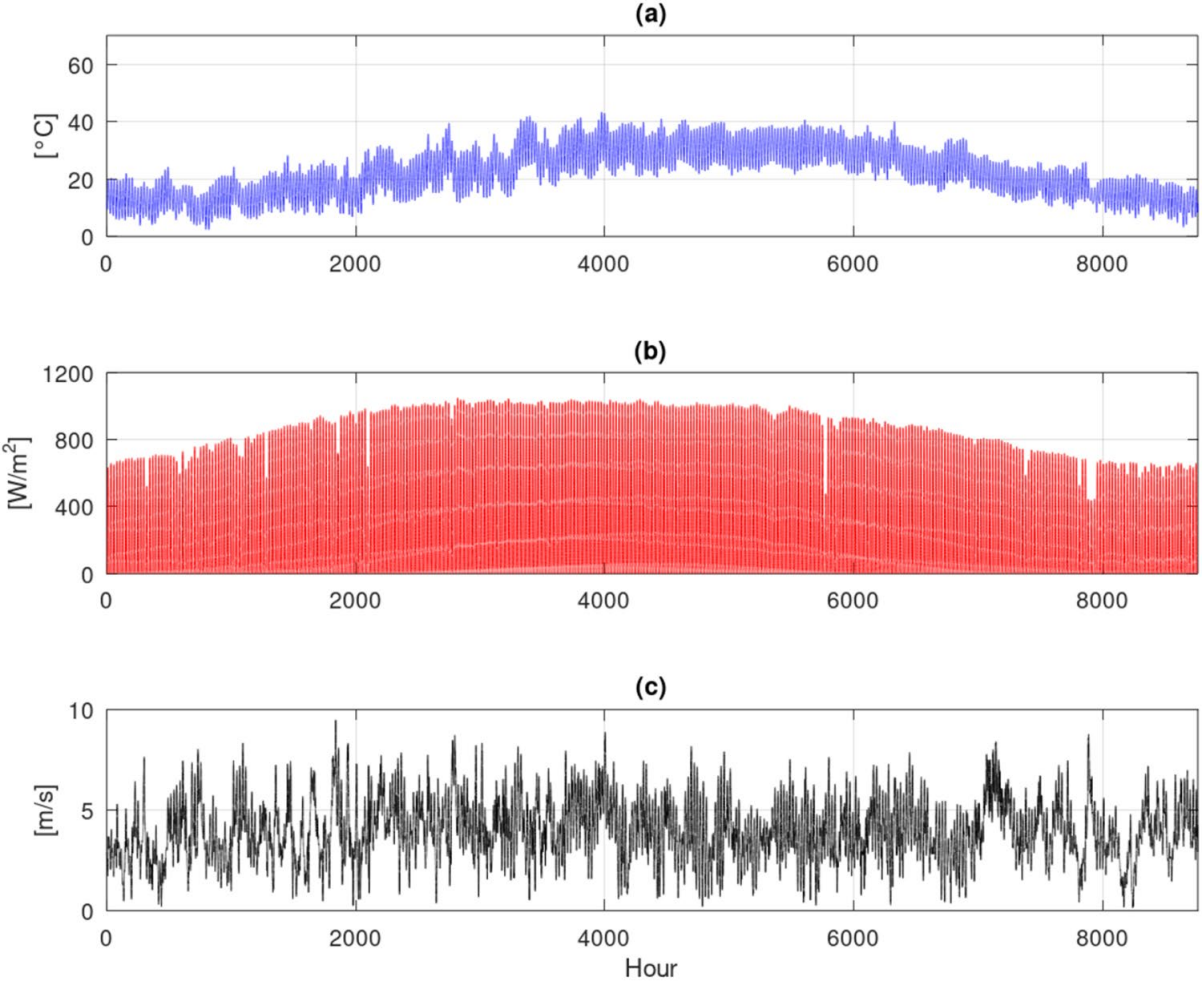
ERA-Land hourly data, (**a**) AAT; (**b**) Solar Radiation; (**c**) wind speed, for location Bahariya Oasis, Egypt.

Figure 2 present the AAT, wind speed, and solar radiation for the location Bahariya Oasis, Egypt. As clearly illustrated in Fig. 2a, the site experiences a pronounced hot summer, which poses a significant climatic challenge for agricultural endeavors. Maximum ambient temperatures consistently exceed 30$\,{}^{\circ }$C, notably surpassing the optimal cultivation range for a multitude of conventional crops^28^. For instance, the recommended temperature range for tomato cultivation typically falls between 20 and 30$\,{}^{\circ }$C^29^. Conversely, during winter, temperatures frequently drop below 5$\,{}^{\circ }$C, which similarly falls outside the suitable range for continuous, year-round crop production. Given these extreme temperature fluctuations, it is evident that a greenhouse equipped with active cooling and heating systems is required to maintain the necessary thermal environment for sustainable agricultural practices in this challenging climate.

### Soil subsurface temperature profile

**Fig. 3:**
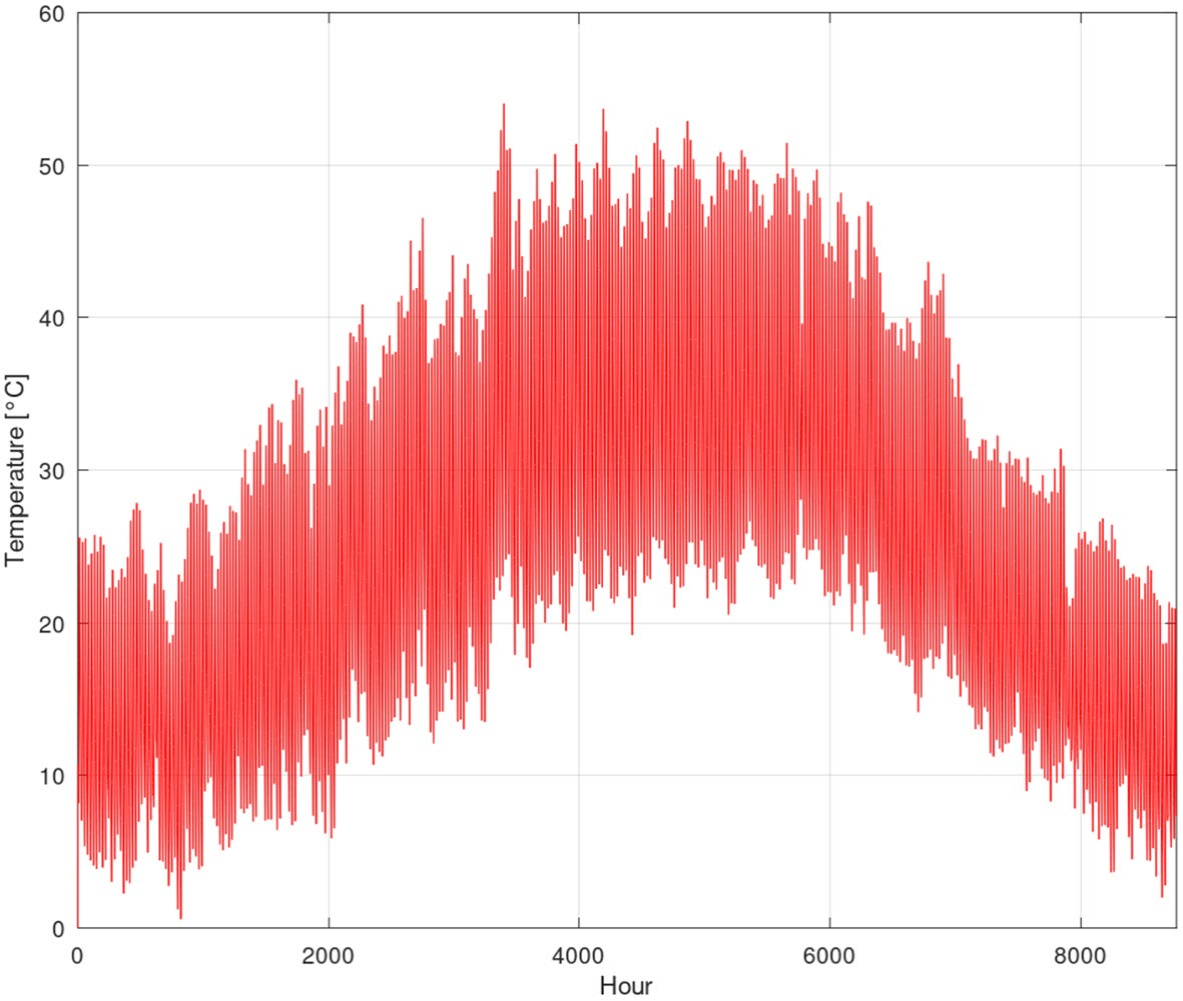
ERA-Land hourly LST for the location Bahariya Oasis, Egypt.

**Fig. 4:**
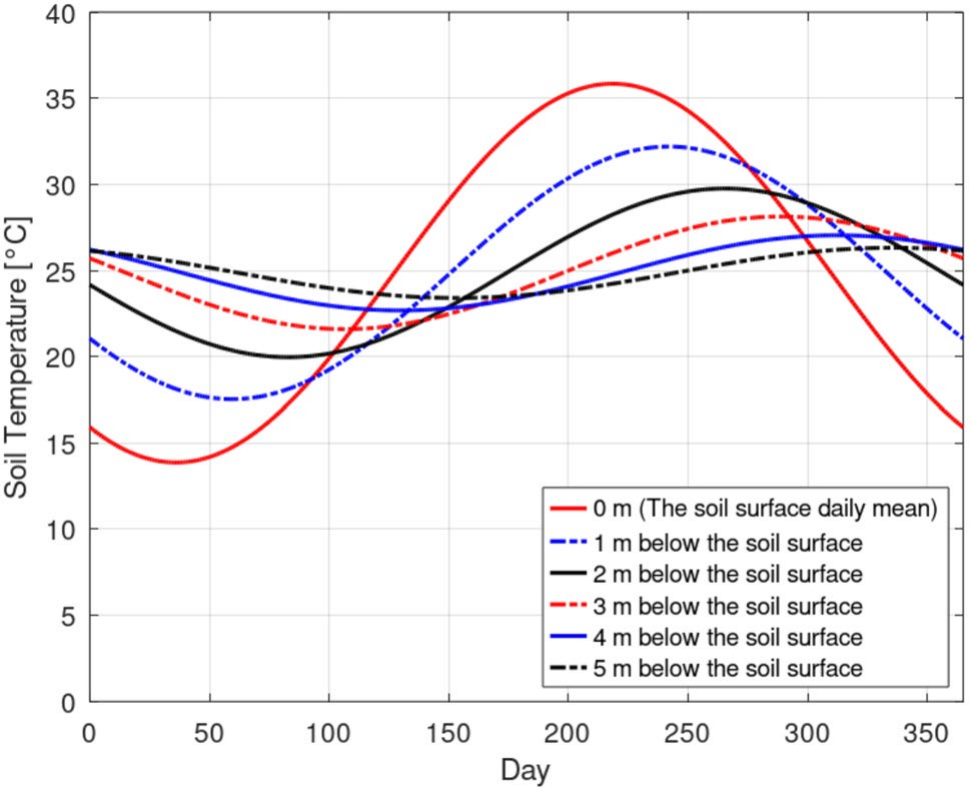
Modeled sub-surface temperature profile with depth for location Bahariya Oasis, Egypt.

Following the approach detailed in Ref.^7^ our analysis of ERA5-Land LST annual data of the year 2024, shown in Fig. 3, yielded an annual mean land surface temperature $T_{mean}$ of 24.86$\,{}^{\circ }$C and a surface temperature fluctuation amplitude $\Delta T_{surface}$ of 10.98$\,{}^{\circ }$C. Incorporating these values into the aforementioned Eq. (7), the predicted temperature profile with respect to depth for the specific climatic conditions of Bahariya Oasis is presented in Fig. 4. The results clearly demonstrate that soil temperature fluctuations are significantly damped with increasing depth. Notably, as we approach a depth of 3 m, the soil temperature becomes remarkably stable, exhibiting a minimal fluctuation of only 2$\,{}^{\circ }$C year-round. This observed thermal stability suggests that an installation depth between 3 and 5 m offers a favorable opportunity for optimal geothermal potential. Exploring greater depths beyond 5 m would incur increased excavation costs without providing a substantial additional geothermal advantage. This finding aligns consistently with several studies^4,7–9^. Therefore, in the current investigation, an installation depth of 4 m was selected for the EAHE system.

### Greenhouse with no temperature control

**Fig. 5:**
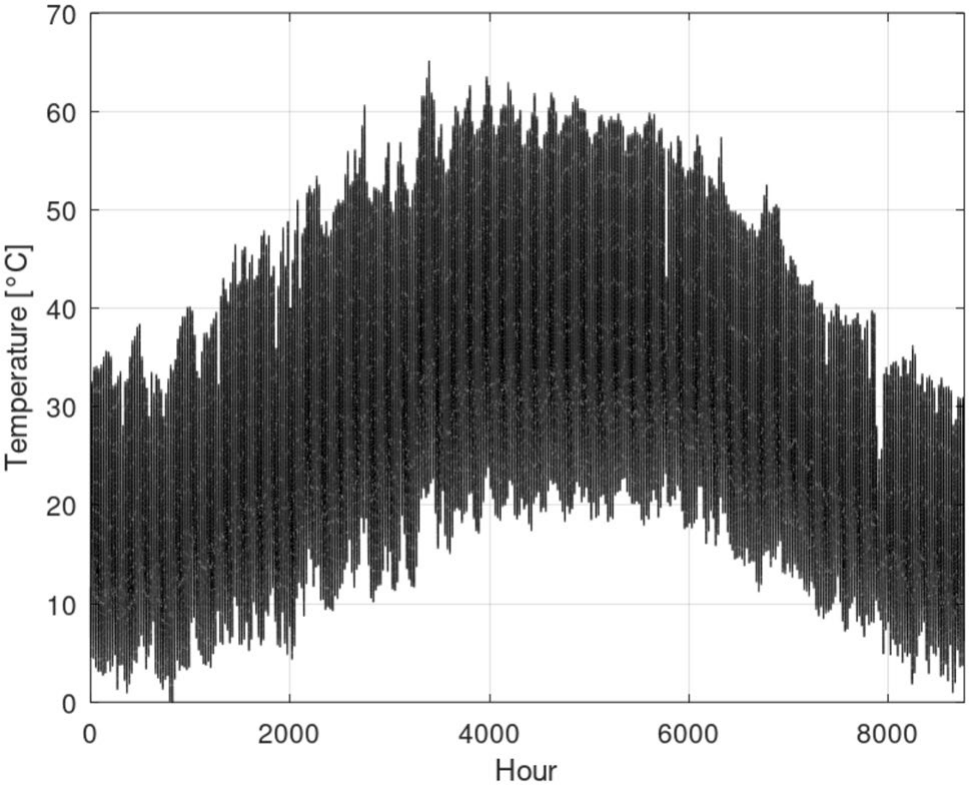
Hourly greenhouse temperature without temperature control.

Figure 5 illustrates the simulated internal temperature of the greenhouse under a scenario with no active temperature control and no ventilation. To model this condition, the ventilation rate (represented by $\dot{Q}_{E}$) was set to zero. As depicted, the internal temperature during the summer months frequently exceeds 60$\,{}^{\circ }$C, while dropping to below 10$\,{}^{\circ }$C in winter. Such extreme temperature fluctuations are entirely intolerable for viable crop cultivation. One potential strategy to mitigate these harsh conditions is to introduce ventilation using ambient air. However, as shown in Fig. 2a, the ambient air temperature during summer frequently surpasses 30$\,{}^{\circ }$C, at times approaching 40$\,{}^{\circ }$C. On the other hand, in winter, ambient temperatures consistently fall below 5$\,{}^{\circ }$C. This demonstrates that direct ventilation with ambient air would not be an effective strategy; instead, it would impose an additional cooling load in summer and an increased heating load in winter.

In contrast, as discussed in the preceding section, low-enthalpy geothermal energy presents a promising opportunity. The subsurface temperature profile, particularly at depths up to 3 m, maintains a remarkably constant and moderate temperature year-round, as seen in Fig. 4. This stability makes it an ideal heat source for winter heating and a heat sink for summer cooling, offering a far more effective and sustainable approach to greenhouse climate control in this environment.

### Greenhouse with low-enthalpy geothermal energy EAHE integration

**Fig. 6:**
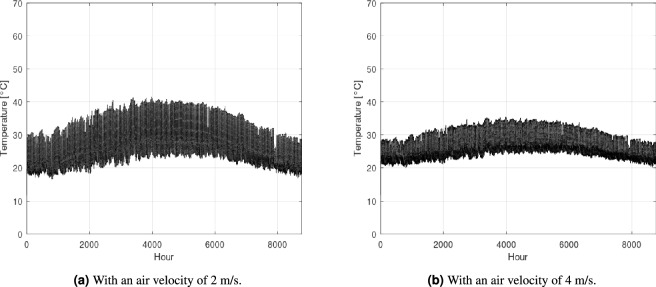
Greenhouse temperature 10 pipes integration with different air velocities.

Similar to the strategy employed by Hegazy et al.^4^ in their study in Hurghada, our investigation began by integrating 10 EAHE pipes operating in parallel into the greenhouse system. This initial configuration was chosen due to the comparable arid climatic conditions between the two study locations in Egypt. As illustrated in Fig. 6a the internal greenhouse temperature with this 10-pipe configuration reached approximately 40 $\,{}^{\circ }$C during summer, while maintaining temperatures near 20 $\,{}^{\circ }$C in winter. This indicates that the 10-pipe EAHE system effectively provided the necessary heating during colder periods. However, the summer temperatures suggest that additional cooling capacity is required to bring the internal conditions closer to the optimal range for crop cultivation. This initial finding underscores the inherent flexibility of EAHE systems in delivering both heating and cooling functions.

To address the observed temperature deviations, particularly during summer, we considered either of two strategies. Firstly, given that the considered air velocity within the pipes is 2 m/s, the air flow rate could be doubled by increasing the air velocity from 2 to 4 m/s. Alternatively, the number of operational EAHE pipes could be doubled (i.e. 20 pipes). While this latter option promises enhanced thermal control, it would inherently incur a higher initial capital cost due to increased excavation and material expenses. Following the implementation of a speed of 4 m/s, as depicted in Fig. 6b, the summer temperatures successfully dropped to approximately 35$\,{}^{\circ }$C, while winter temperatures consistently remained closely above 20$\,{}^{\circ }$C. This demonstrates that the EAHE configuration effectively brought the internal greenhouse temperature close to the ideal range year-round. It is worth noting that to further drop the greenhouse temperature, more EAHE pipes could be implemented. However, that might present practical challenges of excavation and installation.

As shown in Fig. 6b , the greenhouse temperature remains within the ideal range of 20–30$\,{}^{\circ }$C^29^ for most of the year. However, it can rise to 35$\,{}^{\circ }$C during the summer months. While this temperature is outside the optimal range, it may be tolerable for certain crops^30^. To avoid heat stress and ensure a safer growing environment, a recommended strategy is to cultivate heat-tolerant crops^31^, such as okra^32^, during the summer. This highlights how a pre-installation assessment of the geothermal system can inform not only the system design but also the seasonal crop choices throughout the year. If year-round cultivation is not a priority, the greenhouse could be shut down during the summer months. This time could then be used for maintenance and preparing the greenhouse for the next growing season.

**Fig. 7:**
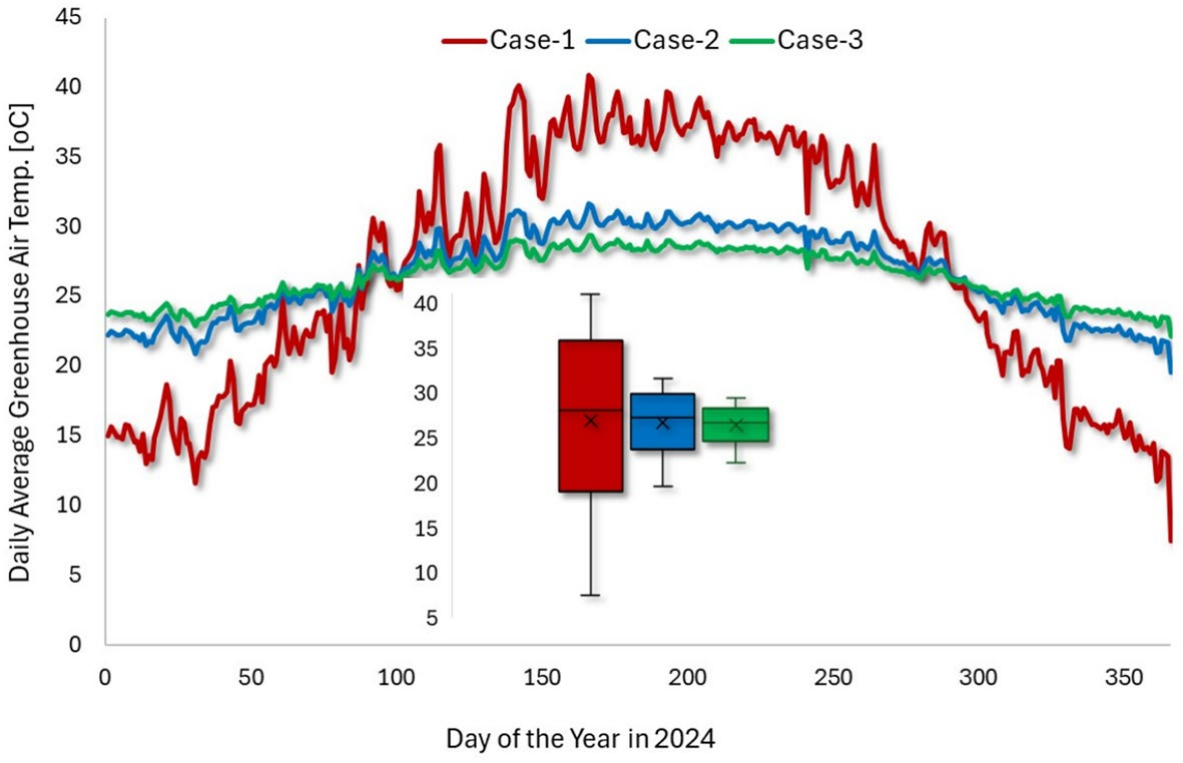
Cases comparison of daily average greenhouse temperature on an annual cycle (Case-1: greenhouse no temperature control; Case-2: 10 pipe air velocity of 2 m/s; Case-3: 10 pipe air velocity of 4 m/s.

In Fig. 7, we have plotted the daily greenhouse temperature average for all the simulated cases. As shown in Fig. 10, in Case-2 and Case-3 a consistent greenhouse daily average temperature was maintained within a range of 20–32$\,{}^{\circ }$C on diurnal scales and 25–27$\,{}^{\circ }$C on annual scales, irrespective of external seasonal variations. This stable thermal environment is highly conducive to continuous, year-round crop cultivation, enabling continuous agricultural production and a steady income stream. These findings highlight the significant potential of protected agriculture for sustainable and economically viable desert farming.

### Diurnal temperature regulation analysis

**Fig. 8:**
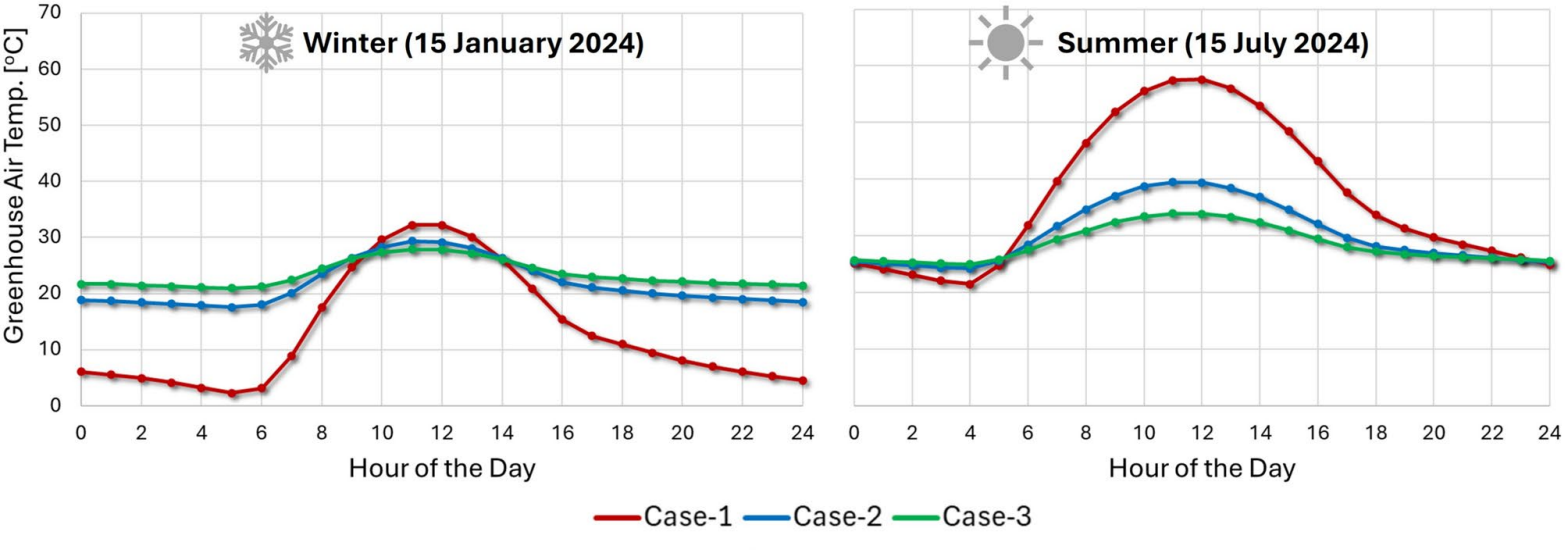
Cases comparison of daily average greenhouse temperature on an annual cycle (Case-1: greenhouse no temperature control; Case-2: 10 pipe air velocity of 2 m/s; Case-3: 10 pipe air velocity of 4 m/s.

To further investigate the effectiveness of the two proposed temperature regulation strategies, we analyzed the diurnal temperature variations within the greenhouse on two representative days: July 15th (summer) and January 15th (winter). These specific days illustrate the dynamic performance of the regulation strategies (i.e. Case-2 and Case-3) compared to the control case (no regulation) (i.e. Case-1). The diurnal temperature profiles, as depicted in Fig. 8, support the findings from the annual analysis in Fig. 6, which indicated that the greenhouse interior temperatures generally remain within an acceptable range for most of the year (8760 h). However, a more detailed examination of these diurnal variations reveals an interesting observation. Specifically, in the absence of any temperature regulation, the greenhouse environment naturally maintains temperatures within the acceptable range of 20–30$\,{}^{\circ }$C^29^ for certain periods. During the winter day (January 15th), this occurs between 08:00 and 14:00. In the summer (July 15th), the acceptable range is maintained from 00:00 to 06:00 and again from 20:00 to 24:00. This suggests that the operation of the EAHE fan system, which circulates air through the pipes, is not continuously required. Consequently, implementing a simple on-off control mechanism for the EAHE fan system could lead to energy savings by preventing unnecessary operation. While the power consumption of these fans is relatively low, as confirmed in Ref.^4^, and could readily be met by solar power^4^, optimizing their operation through such a control strategy would enhance the overall energy efficiency of the greenhouse system. As Fig. 8 demonstrates, the geothermal system effectively regulates the greenhouse temperature throughout the day and night, regardless of the season. By utlizing the stable subsurface soil temperature, the system provides both the necessary cooling during the summer and heating during the winter, maintaining a consistent temperature over the entire 24-h cycle of the warmest and coldest days.s.

## Future work

Based on our findings, several key areas for future research have been identified. First, a detailed economic analysis is essential. This analysis should fully account for the costs of excavating, installing, operating, and maintaining the system. Secondly, since this study did not include a crop inside the greenhouse, future research should investigate the system’s performance with a crop present. This would provide a more complete understanding of the system’s behavior, particularly during the full-growth stage. Another area for future study is the integration of passive cooling strategies, such as shading, to manage high solar loads and improve overall system efficiency.

## Conclusion

This study demonstrated the efficacy of the EAHE low-enthalpy geothermal energy system for greenhouse climate control in arid environments, specifically utilizing the challenging conditions of Bahariya Oasis, Egypt, as a case study. Our initial climatic analysis confirmed the challenging climate in these regions, making unconditioned or passively ventilated greenhouses unsuitable for consistent crop production. A key outcome of our integrated methodology was the successful implementation of ERA5-Land data for subsurface soil temperature profiling, providing crucial design inputs in areas where traditional meteorological data are scarce.

The research’s central contribution lies in validating the remarkable thermal stability of subsurface soil at depths beyond 3 m—firmly establishing its potential as a reliable, year-round heat source and sink. Our simulations quantified EAHE performance: while a 10-pipe configuration effectively handled winter heating, it required augmentation for summer cooling. We conclusively showed that optimizing the EAHE by increasing air velocity from 2 m/s to 4 m/s successfully modulated internal greenhouse temperatures to near-optimal ranges (below 35$\,{}^{\circ }$C in summer, above 20$\,{}^{\circ }$C in winter). This achievement underscores the EAHE’s dual capacity for both heating and cooling in extreme climates. While effective, the study also provided practical insights into the limitations of solely scaling EAHE systems for peak summer loads, given constraints like high initial capital costs. Additionally, based on the findings of this study, several promising directions for future research have been identified.

## Data Availability

The data sets used and analyzed during the current study are available from the corresponding author upon reasonable request.
